# Ciliopathy in PCS (MVA) syndrome

**DOI:** 10.18632/oncotarget.5244

**Published:** 2015-08-22

**Authors:** Tatsuo Miyamoto, Shinya Matsuura

**Affiliations:** Department of Genetics and Cell Biology, Research Institute for Radiation Biology and Medicine, Hiroshima University, Hiroshima, Japan

**Keywords:** primary cilium, ciliopathy, spindle assembly checkpoint, mitotic kinase, mitotic kinesin

The spindle assembly checkpoint (SAC) is a surveillance mechanism of faithful chromosome segregation during mitosis. Budding uninhibited by benzimidazole-related-1 (BubR1) plays a central role in the SAC through inhibition of anaphase promoting complex/cyclosome (APC/C) activity until all chromosomes have established proper attachment to the mitotic spindle. Loss-of-function mutations in the *BUB1B* gene, encoding BubR1, cause a rare autosomal recessive disorder: premature chromatid separation (PCS) syndrome (Mendelian Inheritance in Man [MIM] 176430), which is also known as mosaic variegated aneuploidy (MVA) syndrome (MIM 257300) [1]. PCS (MVA) syndrome can also be caused by an intergenic mutation 44-kb upstream of the *BUB1B* gene, which suppresses the transcription level of *BUB1B* [2]. PCS (MVA) syndrome is cytogenetically characterized by PCS in > 50% of metaphase cells as well as the presence of mosaic aneuploidy [2]. Patients with the syndrome show an increased genetic predisposition to Wilms tumor and rhabdomyosarcoma, which may be attributed to the loss of SAC function. Patients also present with cataracts, uncontrollable chronic seizures, Dandy-Walker complex, polycystic kidneys and obesity. These clinical symptoms imply an additional function of BubR1 other than SAC in the context of morphogenesis.

The primary cilium is a hair-like, microtubule-based, nonmotile organelle formed on the surface of quiescent mammalian cells. It receives extracellular information and transmits signals required for cell proliferation, tissue development, and tissue homeostasis. Ciliary dysfunction is causally linked to the group of human disorders known as ciliopathies, which include Bardet-Biedl syndrome, nephronophthisis, Meckel-Gruber syndrome, Joubert syndrome, and Oral-facial-digit syndrome. To date, more than 50 loci associated with ciliopathy have been reported. In addition to these, Baker and Beales proposed that at least 193 diseases in the Online Mendelian Inheritance in Men database (OMIM, http://www.ncbi.nlm.nih.gov/sites/entrez?db=OMIM) can potentially be categorized as ciliopathies [3]. Patients with a ciliopathy show a series of clinical features including polycystic kidneys, polydactyly, obesity, situs inversus, and neuronal and other developmental abnormalities. Overlap of the clinical spectrum of PCS (MVA) syndrome with those of the ciliopathies has led us to explore whether BubR1 is required for primary cilium formation. We found that skin fibroblasts from PCS (MVA) patients exhibit impaired ciliogenesis, thus PCS (MVA) syndrome is a ciliopathy [4]. Interestingly, *BubR1*-knockdown medaka fish also showed ciliopathy-like phenotypes, such as defective cerebellar development and disrupted left-right asymmetry of the embryo. These results indicated that the role of BubR1 in primary cilium formation is conserved among vertebrates [4]. However, little is known about the molecular basis underlying defective ciliogenesis in PCS (MVA) syndrome.

Primary cilium formation and disassembly are tightly regulated in a cell cycle-dependent manner. In the quiescent G0 phase, centrosomes are anchored to the apical plasma membrane, and primary cilia assemble from the basal bodies transformed from the mother centrioles of the centrosome. When quiescent G0 phase cells re-enter the cell cycle, primary cilium disassembly occurs, suppressing inappropriate ciliogenesis during the proliferative phase. This negative regulation of primary cilium formation is required for the release of centrioles from the apical membrane to form a mitotic bipolar spindle in the context of cell proliferation. Recent studies have revealed that a mitotic kinase, polo-like kinase-1 (PLK1), activates a tubulin deacetylase, HDAC6, to destabilize the axonemal microtubules and initiate primary cilium disassembly following growth stimulation [5]. PLK1 phosphorylates various substrates to control mitotic spindle assembly and chromosome segregation, however the molecular mechanism of PLK1-mediated primary cilium disassembly is not well understood. Importantly, it was reported that KIF24, which belongs to the kinesin-13 family along with KIF2A, KIF2B and MCAK/KIF2C, depolymerizes centriolar microtubules to suppress inappropriate ciliogenesis during the proliferative phase [6]. Kinesin-13 proteins do not move along microtubules for intracellular transport but have microtubule depolymerizing activity. PLK1 phosphorylates KIF2A, KIF2B and MCAK/KIF2C to enhance microtubule depolymerizing activity for mitotic progression. Recently, we demonstrated that PLK1 phosphorylates KIF2A at T554 at the mother centriole, to promote its microtubule depolymerizing activity and drive primary cilium disassembly in a growth signal-dependent manner [7]. Together, we concluded that the PLK1-KIF2A axis is defined as a ciliary disassembly pathway coupled with cell proliferation.

We previously found that BubR1 localizes to centrosomes during interphase to inhibit premature activation of PLK1, and that PCS (MVA) syndrome patient cells show constitutive activation of PLK1 throughout the cell cycle. Inhibition of *PLK1* and *KIF2A* in patient cells restores ciliogenesis [7], indicating that aberrant activation of the PLK1-KIF2A axis contributes to the ciliopathy-associated features in PCS (MVA) syndrome. PLK1 and KIF2A are up-regulated in many types of cancer cells. Further studies on the PLK1-KIF2A pathway in primary cilium disassembly will explore therapeutic opportunities against not only the ciliopathies but also various cancers.
